# Translation and silencing in RNA granules: a tale of sand grains

**DOI:** 10.3389/fnmol.2014.00068

**Published:** 2014-07-23

**Authors:** Jerónimo Pimentel, Graciela L. Boccaccio

**Affiliations:** ^1^Instituto LeloirBuenos Aires, Argentina; ^2^Instituto de Investigaciones Bioquímicas Buenos Aires – Consejo Nacional de Investigaciones Científicas y TecnológicasBuenos Aires, Argentina; ^3^Facultad de Ciencias Exactas y Naturales, University of Buenos AiresBuenos Aires, Argentina

**Keywords:** RNA granule, polysome stalling, mRNA silencing *foci*, localized translation, synapse plasticity, processing bodies

## Abstract

The transcriptome at the synapse consists of thousands of messengers encoding several cellular functions, including a significant number of receptors and ion channels and associated proteins. The concerted translational regulation of all these molecules contributes to the dynamic control of synaptic strength. Cumulative evidence supports that dendritic RNA granules and mRNA-silencing *foci* play an important role in translational regulation. Several relevant RBPs – FMRP; FUS/TLS; TDP-43; Staufen; Smaug; Pumilio; CPEB; HuD; ZBP1; and DDX6 among others – form granules that contain dormant mRNAs repressed by multiple pathways. Recent reports indicate that dendritic granules may contain stalled polysomes, and furthermore, active translation may occur in association with RNA granules. Here, we discuss the molecules and pathways involved in this continuum of RNA granules that contain masked mRNAs, mRNAs trapped in inactive polysomes or mRNAs engaged in translation.

“…siempre se interponían varias hojas entre la portada y la mano. Era como si brotaran del libro”

“El número de páginas de este libro es exactamente infinito. Ninguna es la primera; ninguna, la última.”

Several pages always lay between the cover and my hand. It was as if the pages sprouted from within the book. The number of pages in this book is no more or less than infinite. None is the first page, none the last.

Jorge Luis Borges, “*The book of sand*”

## INTRODUCTION

The transcriptome at the synapse comprises thousands of messengers encoding highly diverse proteins, which include an important proportion of the receptors and ion channels that modulate synaptic plasticity. The transcripts for several variants of the voltage-gated ion channels Kv1.1/Kcna1; Kcnab; Kcnama; Cacna; Cacnab; Cancng; Scn2a; Scn4b; 20 GABA receptor subunits and nine different glutamate receptors are present at dendrites and in the vicinity of synaptic contacts (Raab-Graham et al., 2006; Cajigas et al., 2012). The concerted translational regulation of all these channels and receptors, along with additional molecules linked to intracellular signaling, cytoskeleton remodeling, and protein metabolism, among other cellular functions, contributes to the dynamic control of synaptic strength.

The presence of RNA granules at dendrites and synapses has been extensively documented. These higher-order assemblies of mRNAs and proteins are thought as units for mRNA transport and translational regulation. The molecular mechanisms for mRNA repression during granule movement are partially known and involve several RNA-binding proteins (RBPs; Thomas et al., 2014). Dendritic RNA granules may contain stalled polysomes, thus allowing a fast resuming of protein production (Graber et al., 2013). Furthermore, active translation may occur in association with the granules. All this suggests that the collection of dendritic RNA granules is almost a continuum that range from mRNAs masked and silenced at the initiation level – even before the pioneer translation round – to mRNAs stuck in inactive polysomes or engaged in active translation (di Penta et al., 2009; Baez et al., 2011; Tatavarty et al., 2012; Graber et al., 2013; Buxbaum et al., 2014).

## SYNAPTIC mRNA SILENCING *foci*

The mRNA-silencing *foci* are large multimolecular assemblies that contain silent mRNAs in association with repressor factors, including miRNAs and specific RBPs. The mRNA-silencing *foci* are, in general, highly dynamic and their dissolution correlates with translational activation. The processing bodies (PBs) are ubiquitous mRNA-silencing *foci* and specialized assemblies are formed under specific conditions, as for example the stress granules (SGs), which are induced upon cellular stress (Mitchell and Parker, 2014; Thomas et al., 2014). PBs – or related assemblies – were predicted and confirmed to be present in dendrites and synapses. However, they are not archetypical PBs, as shown by independent studies that reported that subsets of PB components co-localize in different granules (Cougot et al., 2008; Zeitelhofer et al., 2008; di Penta et al., 2009). An important observation is that these dendritic PBs respond to neuron activity. *N*-Methyl-D-aspartate receptor (NMDAR) stimulation induces the dissolution of a specific type of PB that contains the Decapping Coactivator Protein 1a (DCP1a), putatively releasing transcripts to allow their translation (Cougot et al., 2008; Zeitelhofer et al., 2008). Recent work identified a novel type of mRNA silencing *foci* that contain the repressor Smaug1/Samd4a and that are different from PBs. These granules termed S-*foci* are specific to neurons and associate to the post-synapse. Like the neuronal PBs that contain DCP1a, the S-*foci* dissolve upon NMDAR activation. The mRNA coding for Calcium/Calmodulin-dependent protein Kinase II α (*CamKII*a), a signaling molecule that is key to synaptic plasticity, is repressed at the S-*foci*, and dissolution of the S-*foci* correlates with increased translation of *CamKII*a mRNA (Baez et al., 2011).

**FIGURE 1:**
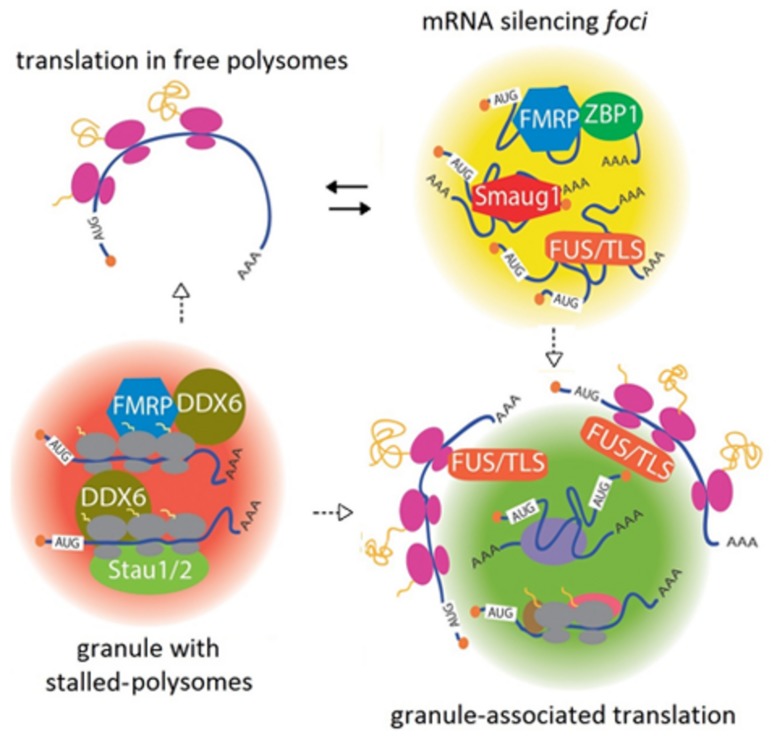
**A continuum between mRNA silencing *foci* and translationally active granules.** Transcripts are repressed in large complexes containing specific RBPs, termed mRNA silencing *foci*. Among other proteins, FMRP, FUS/TLS, ZBP1, and Smaug1/Samd4a form different masking granules. For simplicity, these regulatory factors are depicted together in the same granule. These mRNA-silencing *foci* respond to synaptic activity and reversibly release mRNAs to allow their translation (black arrows). Speculatively, granule reorganization and release of factors may allow translation at their periphery (dotted arrows). Granules with stalled polysomes are found in dendritic granules and may contain FMRP and Staufen molecules. A role for DDX6/RCK/p54/Me31B, which is present in dendritic granules and stalls polysomes in yeast, is speculated (see the text). DDX6/RCK/p54/Me31B would be recruited through the interaction with FMRP and additional RBPs. Dissolution or restructuration of the granules to release stalling factors would allow translation in either free polysomes or granule-associated polysomes (dotted arrows). Granule-associated translation was demonstrated for *ARC/Arg3.1* mRNA and *FMRP* mRNA and might be a common mechanism that also operates in dendritic PBs. The role of FUS/TLS is speculated from data from non-neuronal cells. Translation would occur in the granule periphery, and this may involve reorganization of the mRNA-silencing *foci* or stalled polysome granules.

Another important mechanism for synaptic plasticity is the regulated translation of β-actin mRNA, which affects cytoskeleton remodeling at the post-synapse. By using single-molecule *in situ* hybridization approaches and transgenic animals to visualize the β-actin mRNA *in vivo*, the laboratory of R. Singer recently confirmed that this messenger is present in dendritic granules in a masked state. These granules contain multiple β-actin mRNA molecules and the Zip code Binding Protein 1 (ZBP1). Upon neuron depolarization, the β-actin mRNA is reversibly released along with ribosome subunits, which are similarly masked (Buxbaum et al., 2014; Park et al., 2014). A tempting speculation is that several other transcripts go through masking/unmasking cycles governed by neuron activity and involving specific factors, including in addition to Smaug1 and ZBP1, the RBPs HuD; Fragile X Mental Retardation Protein (FMRP); TAR DNA-binding protein 43 (TDP-43); Fused in Sarcoma/Translocated in Sarcoma (FUS/TLS); Cytoplasmic Polyadenylation Element-Binding protein (CPEB); Pumilio and several PB components (Thomas et al., 2014; **Figure 1**).

The proteins directing the formation of RNA granules are expected to contain oligomerization domains. In connection with this, low complexity regions (LCR) believed to mediate protein self-aggregation are frequent in the RBPs present in granules isolated from neural tissues (Han et al., 2012; Kato et al., 2012). Among other examples, fly Pumilio, which regulates the sodium channel Scn1a, oligomerizes through a QN-rich region. A related pathway involving QN-domains controls the aggregation of CPEB, which regulates the length of the polyA tail of several messengers (Kruttner et al., 2012; Thomas et al., 2014). The dissolution of the mRNA-silencing *foci* or masking granules is governed by signaling pathways downstream of synaptic activation. The unmasking of β-actin mRNA upon depolarization is mediated by MEK1/2, and the dissolution of the S-*foci* upon NMDAR stimulation requires calcium entrance and the activation of the PI3K/mTOR pathway (Baez et al., 2011; Buxbaum et al., 2014). These signal transduction cascades hypothetically affect protein aggregation with direct consequences in granule organization and mRNA repression. RNA granules may dissolve completely to release transcripts or restructure to allow mRNA translation at their periphery, as discussed below (**Figure 1**).

## GRANULES WITH STALLED POLYSOMES

Polysome stalling is a cellular strategy to control the production of proteins specifically required in acute conditions. In a pioneer work, Chapman and Walter (1997) reported that under normal conditions a key stress transcript is trapped in halted polysomes that resume elongation upon cellular stress. The immediate response to synaptic stimulation exploits this mechanism and stalled polysomes present in dendrites are reactivated to allow the rapid production of a number of key proteins. The work by Darnell et al. (2011) greatly contributed to our current knowledge on polysome stalling by FMRP. The interaction of FMRP with polysomes halts translation by unknown mechanisms, which are inactivated upon FMRP phosphorylation or degradation (Darnell et al., 2011). More recently, the laboratory of W. Sossin used a battery of translation blockers and metabolic labeling of proteins *in situ* to demonstrate that a large proportion of dendritic RNA granules contain stalled polysomes, which switch into active translation upon metabotropic receptor stimulation (Graber et al., 2013).

Which protein factors are involved in polysome stalling at dendritic granules? As expected, FMRP is present in these granules, but with a low frequency. Only 10% of them contain FMRP, suggesting the participation of additional RBPs. In fact, 50% of the granules with stalled polysomes contain the double-stranded RBP Staufen 2 (Graber et al., 2013). Both Staufen 1 and Staufen 2 are critical to neuron function, form dendritic RNA granules and associate with polysomes. Indirect evidence suggests that Staufen 1 stalls translation and most likely, Staufen 2 elicits a similar effect (Thomas et al., 2009; Heraud-Farlow et al., 2013). A relevant observation is that Staufen 2 associates with Map1b mRNA granules and this association is interrupted by metabotropic receptor stimulation, which triggers MAP1b mRNA translation. Inhibition of translation initiation does not impair MAP1b mRNA translational activation, thus indicating that MAP1b mRNA is reversibly stalled at elongation, speculatively by the action of Staufen 2 (Lebeau et al., 2011; Graber et al., 2013). In addition to FMRP and Staufen molecules, the PB protein Dead Box Helicase 6 (DDX6/RCK/p54) is likely to help polysome stalling. In a recent study, the laboratory of J. Coller demonstrated that the yeast homolog Dhh1 slows polysomes. Dhh1 directly interacts with ribosome subunits affecting both initiation and elongation and as a consequence of stalling, the transcripts may undergo either decapping or storage (Sweet et al., 2012). Yeast Dhh1, vertebrate DDX6/RCK/p54, and invertebrate Me31B are highly homologous and all these molecules act as translational repressors. In the fly embryo, the maternal transripts *oskar* and *nanos* mRNAs are repressed in unproductive polysomes and this involves Me31B (Presnyak and Coller, 2013). In mammalian neurons, DDX6/RCK/p54 is present in dendritic granules that contain ribosomes (Elvira et al., 2006), and the above observations are compatible with the possibility that these ribosomes are stalled. Furthermore, indirect evidence suggests that this is regulated by neuron activity. A large proportion of the dendritic DDX6/RCK/p54 granules also contain Dcp1a, and granules with DCP1a are affected by NMDA and brain-derived neurotrophic factor (BDNF) with opposite responses. NMDA triggers their dissolution, and BDNF induces their assembly in connection with miRNA-mediated silencing, a pathway that may involve translation stalling (Elvira et al., 2006; Cougot et al., 2008; Huang et al., 2012).

DDX6/RCK/p54/Me31B might be involved in FMRP-dependent polysome stalling, as the *Drosophila* homolog interacts with fly FMRP to repress translation. This interaction is likely to be conserved in mammals, thus providing a mechanism for polysome slowing upon FMRP binding (Barbee et al., 2006). The recruitment of DDX6/RCK/p54/Me31B via additional RBPs that recognize specific transcripts seems likely as well (**Figure 1**). As described above for mRNA-silencing *foci* and masking granules, granules with stalled polysomes may respond to specific signals and dissolve to allow translation. Alternatively, they can rearrange to inactivate or release stalling factors, thus allowing translation at their periphery, as described in the next section (**Figure 1**).

## GRANULE-ASSOCIATED TRANSLATION

A number of recent reports demonstrated that translation occurs in the vicinity of dendritic mRNA granules and that polysomes are associated with PBs in several cell types and organisms. In a seminal work, Krichevsky and Kosik (2001) proposed that dendritic mRNA granules function as “activation cartridges,” switching from a silent state into active translation upon neuron depolarization. Later on, the laboratory of J. Carson tracked microinjected *FMRP* and *ARC/Arg3.1* mRNAs and their encoded proteins using single-molecule approaches in living neurons. As anticipated, the injected mRNAs formed granules and unexpectedly, the polypeptides generated from these transcripts were detected near the granules (Tatavarty et al., 2012). This suggests that the messengers do not diffuse away prior to their translation and probably, the granules reorganize to unmask the mRNAs thus allowing translation. Granule-associated translation occurs either in monosomes or in polysomes, and metabotropic receptor stimulation increases the proportion of translation in polysomes, conceivably through the reactivation of stalled polysomes (Tatavarty et al., 2012; Graber et al., 2013). We propose that translating granules come from a significant rearrangement of masking or stalling granules (**Figure 1**).

Recent work in non-neuronal cells demonstrated that granules containing FUS/TLS may similarly support translation (Yasuda et al., 2013). Yasuda et al. (2013) found that FUS/TLS overexpression or the presence of disease-linked mutations result in large RNP granules. By using *in situ* metabolic labeling of proteins, they found that translation occurs in association with FUS/TLS granules. FUS/TLS form plastic granules at dendrites and the above observations open the possibility that synaptic FUS/TLS granules switch between states of silencing and active translation, involving granule reorganization. Pathogenic mutations on FUS/TLS enhance FUS/TLS aggregation and this may impair granule rearrangement and local production of proteins, thus affecting synaptic plasticity and homeostasis (Ramaswami et al., 2013).

The question of where exactly in the granule translation takes place was approached by high-resolution electronic microscopy of *Drosophila* PBs. The laboratory of I. Davis found that the maternal transcripts *bicoid* (*bcd*) and *gurken* (*grk*) mRNAs associate with embryo PBs with different localizations. Whereas *grk* mRNA concentrates at the edge of the PBs, *bcd* mRNA preferentially localizes at the PB core. This correlates with their translational status, and *grk* mRNA is actively translated whereas *bcd* mRNA is repressed. Moreover, *bcd* mRNA relocates to the PB periphery when its translation is triggered later during development (Weil et al., 2012). More recently, similar findings were reported in mammalian PBs. Using immunoelectron tomography, Cougot et al. (2013) found polysomes and the translation factors eIF4G and eIF4E at the PB periphery. Altogether, these findings collectively suggest that the PB periphery can support active translation, and in addition they may be surrounded by stalled polysomes. As in the case of PBs, the translation associated with dendritic RNA granules would occur in their periphery, where protein folding and subunit assembly would be facilitated by chaperones and cytosolic factors, and relatively low space constrains. By regulated granule reorganization and mRNA relocation, mRNAs would access the translational apparatus, and repressor molecules including polysome-stalling factors would be released (**Figure 1**).

In addition to the above-described translational regulation pathways, the packaging of mRNAs in granules is believed to facilitate their transport, and recent findings in non-neuronal cells suggest that the presence of polysomes may help. Working with a fungal model system, Higuchi et al. (2014) showed that polysomes – but not single ribosome subunits – are transported along microtubules in association with endosomes. Endosomes travel long distances in neurons, hypothetically providing a platform for the transport of granules containing polysomes, either actively translating, or stalled. In connection with these speculations, Staufen molecules associate with polysomes and with membranous organelles, potentially coupling polysome stalling to mRNA transport (Thomas et al., 2009).

## CONCLUDING REMARKS

The putative cross-talk and coordination between the above-described pathways, which may involve the exchange of factors among different granules, remain to be further investigated. Adding complexity, a given transcript may be regulated by multiple networks and in different granules, which may shift between translationally silent or active states, all these providing mechanisms for response diversity. The dynamics of mRNA storage and translation are linked to granule condensation, reorganization, and dissolution. These processes are analogous to phase transitions and depend on posttranslational modifications and physicochemical parameters (Brangwynne, 2013). Further investigation on the regulation of protein self-aggregation will provide the basis for mechanistic links between synapse activity, granule plasticity, and translational regulation.

## Conflict of Interest Statement

The authors declare that the research was conducted in the absence of any commercial or financial relationships that could be construed as a potential conflict of interest.
